# Applying the Project ECHO Model to Support Implementation and Sustainment of Cognitive Behavioral Therapy for Psychosis

**DOI:** 10.1097/CEH.0000000000000511

**Published:** 2023-06-29

**Authors:** Sarah L. Kopelovich, Jennifer Blank, Chris McCain, MacKenzie Hughes, Eric Strachan

**Affiliations:** Department of Psychiatry and Behavioral Sciences University of Washington, Seattle, WA.

**Keywords:** evidence-based psychotherapeutic interventions, consultation, implementation, cognitive behavioral therapy for psychosis, continuing education

## Abstract

**Introduction:**

Project Extension for Community Healthcare Outcomes (ECHO) is a teleconsultation model that leverages technology to sustain specialized interventions in underresourced settings. We present the application of the ECHO model to longitudinal training and consultation for community behavioral health providers learning to deliver cognitive behavioral therapy for psychosis, an evidence-based psychotherapy for individuals with psychotic disorders that has poorly penetrated the US mental health system.

**Methods:**

We analyzed within-group change over practitioners' 6-month ECHO participation cycle using the Expanded Outcomes Framework. We evaluated outcomes associated with participation, satisfaction, knowledge acquisition, performance, patient symptom severity, and functional impairment.

**Results:**

In the first 3 years, the cognitive behavioral therapy for psychosis ECHO Clinics supported 150 providers from 12 community agencies. Forty percent did not complete the 6-month ECHO calendar, most commonly due to separation from their agency. Participants reported high degrees of satisfaction. Declarative and procedural knowledge increased over the 6-month period. Of the 24 providers who received a fidelity review, 87.5% met or exceeded the competency benchmark within the 6-month period. Clinical outcomes reflected reductions in hallucinations, negative symptoms, depression, mania, and functional impairment, but no reductions were detected in delusions, disorganized speech, or abnormal psychomotor behavior.

**Discussion:**

ECHO Clinics offer a mode of providing continuous access to expert instruction, peer-to-peer consultation, and case-based learning that other workforce training models lack. Our evaluation suggests that the ECHO model supports continuous professional development for practitioners, most of whom had indicated inadequate preparation for their role. We observed improved learner and select patient outcomes.

In response to the identified need to support practitioners in the treatment of vexing but treatable health conditions, Project Extension for Community Healthcare Outcomes (ECHO) uses telehealth technology, didactics series by expert practitioners, and case-based learning to facilitate the acquisition of knowledge and enhanced self-efficacy in evidence-based treatments (EBTs).^1^ Developed to address the inaccessibility of treatment for hepatitis C, Project ECHO has since expanded to scores of health conditions in more than 34 countries around the world. Recent legislation in the United States^2^ calls for domestic proliferation of specialty ECHO programs and federal agencies recommend the ECHO model as a means to build health care workforce capacity. Like hepatitis C, psychotic disorders are treatable conditions, yet evidence-based care is largely inaccessible in the United States.^3^ National treatment guidelines recommend multicomponent care, including cognitive behavioral therapy (CBT).^4^ Unfortunately, recent estimates suggest that there are only 15 public behavioral health practitioners trained in CBT for psychosis (CBTp) for every 10,000 Americans with a psychotic disorder, and CBTp training has been conducted in geographically concentrated regions.^5^ Therefore, providers in community behavioral health settings often lack training in the requisite knowledge and psychotherapeutic competencies.^6^

Continuous professional development and practice facilitation are considered critical to competent delivery of a specialized intervention like CBTp.^7^ Developing the knowledge base and associated competencies is enhanced by clinical consultation and learning opportunities,^8^ yet these opportunities are exceedingly rare in practice. Training programs that leverage the ECHO model have potential to improve CBTp access and quality across the United States.

We report on the first ECHO Clinics focused on CBT and psychotic disorders. The clinics launched in February 2017 as a state-funded academic-community partnership to implement CBTp in community behavioral health settings. The ECHO Clinics enabled a new model of longitudinal consultation to support the first CBTp Provider Network in the country. Leveraging the ECHO model credos of “once a participant always a participant” and “All teach, all learn,” the CBTp ECHO Clinics were adopted as a means of fostering a professional community, mitigating therapeutic drift through ongoing professional development, enhancing CBTp funds of knowledge and competencies, and improving outcomes of CBTp consumers. Despite a robust body of literature demonstrating satisfaction, engagement, enhanced knowledge, and therapeutic benefit of ECHO participation,^9,10^ we were interested in ascertaining whether such benefits extend to practitioners learning a psychological treatment for psychotic disorders. Consistent with previous evaluations of ECHO clinics for other complex treatments and conditions,^11,12^ we employed the *Expanded Outcomes Framework*, a well-established framework for evaluating professional development activities developed by Moore et al (Figure 1).^13^

**FIGURE 1.**
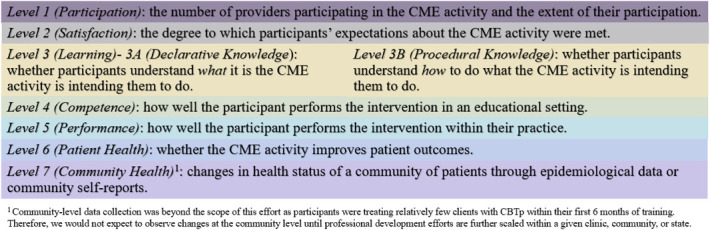
Expanded outcomes framework. Details the seven levels of evaluation of clinical professional development in the *Expanded Outcomes Framework* created by Moore and colleagues.^13^

## METHODS

### CBT ECHO Clinic Description

The CBT ECHO Clinic protocol was based on the ECHO replication model^14^ and previous empirical literature proposing effective elements of longitudinal consultation for CBT interventions, including recommended minimum dose^15^ and opportunities for didactic and case-based learning as well as behavioral rehearsal.^16,17^ The didactic schedule was designed to build on concepts introduced during the workshop training and to align with core elements of CBTp previously identified through expert consensus.^18^ The CBTp ECHO Clinics adhere to the ECHO hub-and-spoke model. The hub team is composed of a clinic coordinator, IT support, and a panel of CBTp trainers with a cumulative 25 years of experience administering the intervention. The CBTp ECHO Clinics are open only to behavioral health providers who have attended a synchronous multiday intensive workshop, during which they receive didactic instruction, experiential activities, and role plays demonstrating the application of CBT to psychotic and mood symptoms. Following participation in the live workshop, participants are expected to attend a minimum of nine of the first 12 ECHO Clinics offered within the first 6 months post workshop (75% attendance requirement). The first 12 ECHO sessions represent the core CBTp ECHO curriculum; subsequent didactics are intended to supplement the core curriculum (Table 1). Thereafter, trainees have an open invitation to attend ECHO Clinics at their own discretion. Contact hours are offered in accordance with state and professional organizational guidelines for teleconsultation, but no other incentives are offered.

**TABLE 1.** CBTp ECHO Clinic Didactic Curriculum

Core Curriculum	Supplemental Curriculum
Case conceptualization	Medication engagement
Session guidelines	Maximizing participation
Home practice	Working with natural supports
CBT for negative symptoms	Thought disorder
CBTp graduation	Substance use and CBTp
Behavioral activation	Command hallucinations
Behavioral interventions for hallucinations (part I)	Third wave CBTp approaches
Behavioral interventions for hallucinations (part II)	The “good enough” therapist
Cognitive restructuring (part I)	Clinician burnout
Cognitive restructuring (part II)	Measurement-based care
Cultivating the relaxation response	CBTp supervision
Cognitive impairments	Addressing problems in CBTp

CBTp, cognitive behavioral therapy for psychosis; CBT, cognitive behavioral therapy.

### CBT ECHO Clinic Protocol

The traditional ECHO session protocol includes introductions of the spokes (prioritizing video callers to reinforce community-building behaviors), introductions of the hub panelists, announcements from spokes and/or hub, a live didactic or role play, and a case consultation. In accordance with the “all teach all learn” credo, questions and recommendations are first elicited from the spoke participants and then are offered by the hub panelists. The clinic coordinator notes clinical recommendations for later dissemination to clinic participants along with resources that were recommended during the clinic. Clinics range from 60 to 90 minutes depending on the complexity of the case presentation or CBTp protocol.

### Clinic Evaluation

The methods and data that were used to evaluate the CBTp ECHO Clinics in accordance with Moore's evaluation framework are described below. This work was reviewed by the Washington State Institutional Review Board and granted exempt determination.

#### Level 1—Participation

Programmatic data were collected February 2017 and August 2021 using iECHO, a web-based data management system operated by the University of New Mexico SuperHub. Data include administrative data, participant attendance, session components, program documents, and awarded CME/CEU/CE credits.

#### Level 2—Satisfaction

We randomly administered a 3-item end-of-session poll using Poll Everywhere to 50% of all clinics to evaluate satisfaction, confidence, and perceived impact on clinical practice. Participants rated their satisfied with the clinics and their degree of benefit on a scale of 1 (Not at all) to 5 (Very great extent) and provided a dichotomous (yes/no) response to “Will this consult affect your work with one or more CBTp clients?” The impact on practice item was also included at 3- and 6-month time points through REDCap (Research Electronic Data Capture^19,20^).

#### Level 3A—Learning: Declarative Knowledge

To assess fund of knowledge of CBTp, 1 multiple-choice “Test Your Knowledge” question was administered following each of the initial 12 core didactics. Scores were coded dichotomously (pass/fail).

#### Level 3B—Procedural Knowledge

Participants were asked to rate their CBTp skill level across 20 CBTp techniques on a 5-point Likert scale (1–minimal; 5–advanced). Example items include “Teaching clients how to reduce distress by evaluating inaccurate or unhelpful thoughts” and “Working collaboratively with clients to develop a list of concerns that cause distress and/or interfere with life goals.” Ratings were totaled for a score range of 20 to 100, with higher scores indicating increased skill in providing CBTp. This self-assessment was administered at preworkshop, postworkshop, and 6-month follow-up.

#### Levels 4 and 5—Competence and Performance

In accordance with national recommendations for establishing CBTp competencies,^21,22^ participants were asked to submit session audio to enable the hub team to assess the degree to which CBTp was being administered to clients with fidelity to the treatment model. Sessions were rated with the Revised Cognitive Therapy for Psychosis Adherence Scale (R-CTPAS),^23^ a psychometrically validated fidelity scale designed to assess adherence and competency across therapeutic activities considered to be core components of CBTp. The scale includes items related to general features of the session (eg, agenda setting, home practice review, and home practice assignment) as well as specific features of a session that correspond to phases of treatment (eg, psychoeducation and engagement, assessment, intervention, skill generalization). Participants were given an overall rating on a scale of 1 to 5; ratings of three or higher are considered to meet competence in CBTp.

#### Level 6—Patient Health

To track patient outcomes, CBT ECHO participants administered the DSM-5 Clinician Rated Dimensions of Psychosis Symptom Severity Scale (CRDPSS)^24^ and the Social and Occupational Functioning Assessment Scale (SOFAS) to their clients.^25^ Both measures are recommended by the DSM-5 task force and as ways of monitoring progress in the CBTp Stepped Care model.^26^ The CRDPSS is a dimensional (0–not present, to 4–present and severe) scale designed to assess 8 symptom domains associated with psychotic disorders, such as hallucinations, delusions, disorganized speech, abnormal psychomotor behavior, negative symptoms, depression, mania, and cognitive functioning. We omitted the cognitive functioning item as trainees were not credentialed to obtain valid and reliable measures of cognitive functioning. The SOFAS is a clinician-rated measure of functioning scored on a scale of 1 (grossly impaired) to 100 (excellent functioning), with benchmarks provided at each multiple of 10. Deidentified patient assessment data were entered into a data dashboard that was mutually accessible to the ECHO participant and the research team. Complete data were defined as 2 or more administrations of a measure, including 1 at baseline and at least 1 at follow-up. For those who completed a given measure at multiple time points, the final time point was defined as “follow-up” and used in analysis.

## RESULTS

During the observation period, 187 behavioral health providers participated in a CBTp workshop. Thirty-seven participants (19.8%) were ineligible to participate in a CBTp ECHO Clinic by virtue of their role as an agency administrator or manager.

### Level 1—Participation

**FIGURE 2.**
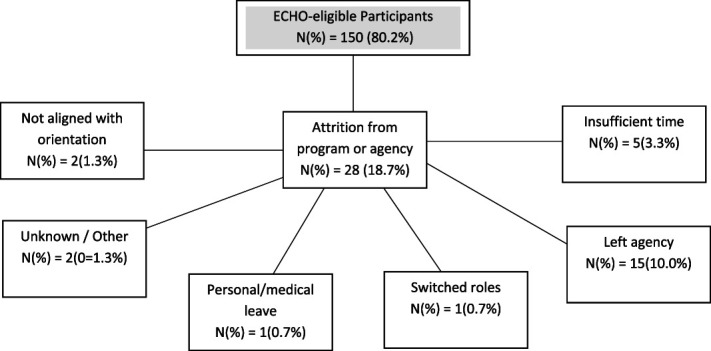
Consort diagram of CBTp trainee. Consort diagram indicating trainees' reasons for discontinuing participation and the number of trainees who discontinued for each identified reason. CBTp, cognitive behavioral therapy for psychosis

One hundred fifty eligible clinicians attended at least 1 ECHO clinic (80.2%; see Figure 2). ECHO participants were employed by 12 agencies across 10 counties in the state. Demographics are reported in Table 2. Most (56.2%) reported inadequate or no training in CBT in their programs of study.

**TABLE 2.** CBTp Trainee Demographics

	N	%
Role		
Supervisor	13	11.4
Clinician	101	88.6
Gender		
Male	39	29.1
Female	94	70.1
Other	1	0.7
Race/ethnicity		
African American	6	5.2
Asian	5	4.3
Caucasian/White	82	70.7
Latino/Hispanic	13	11.2
Multiracial	9	7.8
Other	1	0.9
Highest degree*		
High school	5	4.3
4-y college	15	12.8
Masters	97	82.9
	Mean	SD
Age, y	39.2	11.0
Years at agency*	3.8	5.1
Years providing psychotherapy	5.5	6.9
Years of experience with psychosis	6.2	7.4
Years providing CBT*	4.7	6.3

CBTp, cognitive behavioral therapy for psychosis; CBT, cognitive behavioral therapy.

The training hub hosted 217 CBTp ECHO Clinics between February 2017 and October 2021, facilitating 60 unique didactics and 146 case presentations. Clinic attendance ranged from 2 to 35 trainees (M = 12.9, SD = 4.6). Inclusive of providers who dropped out of the training program prematurely or who paused their participation, trainees attended between 1 and 70 clinics (M = 12.5, SD = 11.1), generating a cumulative 3428 continuing education hours. Sixty percent of trainees (*n* = 90) met the initial 6-month attendance requirement. Of those who did not meet attendance requirements, the most common reason was separation from their agency.

### Level 2—Satisfaction

Trainees reported high degrees of satisfaction immediately following clinics, with a mean rating of 4.2 out of 5 (SD = 0.4). Approximately 550 responses were collected to the end-of-session forced choice question “Will this consult affect your work with one or more CBTp clients?” with 97% of responses endorsing this statement. This postclinic question was administered after nearly half of all ECHO clinics (*n* = 95), with a response rate of approximately 45%. Trainees reported having benefitted from ECHO clinics at both 3- and 6-month points, with a significant increase in self-report ratings from 3-month follow-up (M = 3.52, SD = 1.00) to 6-month follow-up (M = 3.74, SD = 1.04); *t*(57) = −2.09, *P* = .041.

### Level 3A—Declarative Knowledge

We collected 58 responses to the postclinic “Test Your Knowledge” questions following the 12 core didactics (37% response rate). The correct response rate was 73.2% (range = 33.3% to 100%). Trainees scored the lowest on an initial didactic on the topic of case conceptualization and scored the highest on later didactics related to working with clients with cognitive impairments and wellness planning. The temporal trend toward higher pass rates suggests that trainees' understanding of CBTp principles and techniques improved over the course of the ECHO series.

### Level 3B—Procedural Knowledge

To assess whether ratings changed over time, we conducted a pairwise comparison. Mauchly test of sphericity indicated a violation of the assumption of sphericity (X^2^(2) = 7.886, *P* = .019). We therefore applied Greenhouse and Geisser (1958) correction. We detected a significant main effect of time (*F*(1.57, 40.93) = 29.89, *P* < .001; η_p_^2^ = .54), such that participants of both clinics perceived their skills to have improved significantly from pretraining to posttraining and remained significantly higher than baseline at 6-month follow-up. To assess for training effects, we ran Levene test to compare 6-month follow-up scores between those who did and did not meet attendance expectations. On average, completers' skills were significantly greater (M = 65.33, SD = 15.48) than those of noncompleters (M = 53.44, SD = 12.73), *t*(28) = −2.02, *P* = .041; *d* = −.81, 95% CI [−1.61, 0.01].

### Levels 4 and 5—Competence and Performance

A minimum of one CBTp session fidelity review was offered to each trainee who partook in both the live CBTp training workshop and subsequent ECHO clinics. Twenty-four trainees received fidelity reviews, of which 21 (87.5%) met or exceeded the competence threshold within the 6-month training program.

### Level 6—Clinical Outcomes

Trainees were expected to administer CBTp to at least 1 client on their caseload during the 6-month training period. Of those trainees who completed both the 3-month and 6-month posttraining survey (*n* = 61), 53 trainees (86.9%) had initiated CBTp with at least 1 client. Number of clients treated by the 6-month follow-up ranged from 1 to 50 (M = 5.21, SD = 8.48). Complete CRDPSS and SOFAS data were available for 24 and 16 clients, respectively (Table 3). Severity of clients' hallucinations, negative symptoms, depression, and mania were reduced from baseline to follow-up. Disorganized speech and abnormal psychomotor behaviors were scored as minimally severe at both baseline and posttreatment, with no significant changes observed between time points. We detected a statistically significant improvement in functioning on the SOFAS.

**TABLE 3.** Client Outcomes

	Baseline	Posttraining	*t*	Two- sided *P*	Cohen's *d* [95% CI]
	M (SD)	M (SD)			
CRDPSS (N = 24)*					
Hallucinations	3.29 (.86)	2.67 (1.13)	2.90	.008*	.59 [0.15, 1.02]
Delusions	2.54 (1.41)	1.92 (1.32)	1.76	.092	.36 [−0.06, 0.77]
Disorganized speech	.83 (1.05)	.54 (.93)	1.66	.110	.34 [−0.08, 0.75]
Abnormal psychomotor behavior	.54 (1.02)	.50 (.83)	.21	.833	.04 [−0.36, 0.44]
Negative symptoms	1.58 (1.28)	1.04 (1.12)	2.25	.034*	.46 [0.03, 0.88]
Depression	2.46 (1.10)	1.33 (1.09)	4.05	<.001**	.83 [0.35, 1.29]
Mania	1.00 (1.18)	.50 (.88)	2.22	.037*	.45 [0.03, 0.87]
SOFAS (*N* = 16)†	40.56 (10.72)	51.81(12.67)	−3.9	.001**	−.99 [−1.58, −0.37]

* CRDPSS is the Clinician Rated Dimensions of Psychosis Symptom Severity Scale. Lower scores reflect less symptomatology.

† SOFAS the Social and Occupational Functional Assessment Scale. Higher scores reflect higher functioning.

CI, confidence interval.

*P < .05; **P < .001.

## DISCUSSION

ECHO Clinics offer a mode of providing continuous access to expert instruction, peer-to-peer consultation, and case-based learning that other workforce training models lack. As ECHO programs proliferate internationally and are recommended by governmental bodies,^27^ program evaluation and research on ECHO programs are needed to establish best practices for diverse ECHO participants and focal problems. We describe trainee outcomes for the first teleconsultation clinic that applied the ECHO model to a cognitive behavioral treatment and to psychotic spectrum disorder treatment. We report here on the inaugural CBT ECHO Clinic model and participant and patient outcomes using the Expanded Outcomes Framework to facilitate replication and refinement of this model of professional development. Principal advantages of the ECHO model are the ability to reach mental health care practitioners who do not readily have access to professional development in evidence-based interventions and sustain their access over time. We were able to offer longitudinal support, conferring a total of 3428 continuing education hours, to more than 150 behavioral health providers across 12 community behavioral health agencies, most of whom had indicated inadequate preparation for their role. This is notable for 2 reasons. First, it affirms the importance of workforce development for practitioners working with individuals with psychosis in community settings. Second, it provides evidence that our clinics were successful in targeting those practitioners who may be most in need of professional development.

We used several data collection methods throughout the consultation period to facilitate program evaluation, including active monitoring of attendance, live participant polling, and routine survey administration. Multimodal data collection facilitated quality improvement efforts. Specifically, we were able to tailor and develop new didactics in response to trainee demand. Trainees consistently endorsed that the clinical utility of ECHO sessions and clinic satisfaction for our ECHO Clinics were consistent with the ECHO replication literature.^10,28–31^ That said, only 60% of trainees met the 75% attendance requirement. Although this posed a challenge to data collection, it was not unexpected. Trainees were employed in high-volume, publicly funded settings, which commonly experience high caseloads and staff turnover.^32^ Among those trainees who met the minimum attendance requirement, we observed significant gains in measures of procedural CBTp knowledge. Although clinical outcome data were limited, we found that many of the clinical targets for which CBTp is indicated—namely, hallucinations, mood symptoms, and negative symptoms^33^—did improve over the course of the 6-month training program. Conversely, clinical data did not support statistically significant improvements in delusions, for which CBTp is typically effective.^34^

### Limitations and Future Directions

Many of the challenges we observed among our sample are common to EBT implementation efforts and are well-addressed in recently published CBTp implementation guides.^22,35^ As the data reported here represent a program evaluation, the absence of a control group limits our capacity to draw inferences related to how our trainee and client outcomes compare with other consultation approaches. That said, the observed improvements among most focal psychotic and mood symptoms suggests that clients were benefitting from the services they were receiving. Consistent with the previous literature evaluating EBT implementation in community health settings,^36^ many clinicians in our sample faced significant hardships related to client recruitment and retention, audio recording sessions, and protected time for both continuing education activities and delivering psychotherapy. As a result, missing data are a notable limitation of the current evaluation. We encountered attrition among our sample of behavioral health care practitioners in the first 6 months post workshop. Although our attrition rate is consistent with national data on workforce turnover in community behavioral health settings,^37^ we cannot discount the threat of attrition bias to the validity of our findings.

Our evaluation demonstrates that the ECHO model offers a viable model of longitudinal support to practitioners newly learning an evidence-based psychological treatment for Serious Mental Illness. However, sustaining EBTs in routine care settings requires more than access to continuing professional development and clinical supports. Both internal factors external factors are implicated in the long-term sustainment of EBTs.^38^ We find support for use of Project ECHO as one component of a CBTp sustainment plan. We find particular value in the fact that ECHO Clinics can enable provider-to-provider and expert-to-provider learning across health care settings. We recommend a randomized clinical trial to systematically evaluate trainee and client outcomes associated with ECHO-facilitated longitudinal consultation compared with a consultation as usual approach.

**Lessons for Practice**

■ The teleconsultation model of Project ECHO is a viable means of delivering relevant and satisfactory statewide professional development in psychotherapy to community behavioral health practitioners.
■ Constraints inherent to community mental health work (ie, high staff turnover rates; large, diagnostically heterogeneous caseloads; and limited technological resources) also affect the degree to which trainees can fully participate in longitudinal professional development activities.
■ Further research is needed to investigate the effects of ECHO versus traditional consultation methods on provider training process, retention, trainee outcomes, and clinical outcomes for clients served.

## References

[1] Arora S Geppert CMA Kalishman S Academic health center management of chronic diseases through knowledge networks: project ECHO. Acad Med. 2007;82:154–160. 17264693 10.1097/ACM.0b013e31802d8f68 PMC3855463

[2] Wicklund E. New AMA Policy Supports Project ECHO, CPAP Telemedicine Programs. Newton, MA: mHealth Intelligence. 2019. Available from. https://mhealthintelligence.com/news/new-ama-policy-supports-project-echo-cpap-telemedicine-programs. Accessed July 5, 2019.

[3] Interdepartmental Serious Mental Illness Coordinating Committee. The Way Forward: Federal Action for a System that Works for All People Living with SMI and SED and Their Families and Caregivers. Rockville, MD: Substance Abuse and Mental Health Services Administration; 2017. Available from. https://www.samhsa.gov/sites/default/files/programs_campaigns/ismicc_2017_report_to_congress.pdf

[4] Keepers GA Fochtmann LJ Anzia JM . The American Psychiatric Association practice guideline for the treatment of patients with schizophrenia. Am J Psychiatry. 2020;177:868–872. 32867516 10.1176/appi.ajp.2020.177901

[5] Kopelovich S Nutting E Blank J . Preliminary point prevalence of Cognitive Behavioral Therapy for psychosis (CBTp) training in the U.S. and Canada. Psychosis. 2021;14:344–354.

[6] Roth AD Pilling S. Using an evidence-based methodology to identify the competences required to deliver effective cognitive and behavioural therapy for depression and anxiety disorders. Behav Cogn Psychother. 2008;36:129–147.

[7] Hardy KV Espil FM Smith CL Training early psychosis community clinicians in CBT for psychosis: implementation and feasibility. Early Interv Psychiatry. 2021;15:697–704. 32583602 10.1111/eip.13010

[8] Shafran R Clark DM Fairburn CG Mind the gap: improving the dissemination of CBT. Behav Res Ther. 2009;47:902–909. 19664756 10.1016/j.brat.2009.07.003

[9] Zhou C Crawford A Serhal E . The impact of project ECHO on participant and patient outcomes: a systematic review. Acad Med. 2016;91:1439–1461. 27489018 10.1097/ACM.0000000000001328

[10] McBain RK Sousa JL Rose AJ Impact of project ECHO models of medical tele-education: a systematic review. J Gen Intern Med. 2019;34:2842–2857. 31485970 10.1007/s11606-019-05291-1 PMC6854140

[11] Arora S. Project ECHO: democratising knowledge for the elimination of viral hepatitis. Lancet Gastroenterol Hepatol. 2019;4:91–93. 30647014 10.1016/S2468-1253(18)30390-X

[12] Yennurajalingam S Amos CE Weru J Extension for community healthcare outcomes-palliative care in Africa program: improving access to quality palliative care.J Glob Oncol. 5, 2019:1–8. 10.1200/JGO.19.00128 PMC6776016 31335237

[13] Moore DE Jr Green JS Gallis HA. Achieving desired results and improved outcomes: integrating planning and assessment throughout learning activities.J Contin Educ Health Prof. 2009;29, 1–15. 19288562 10.1002/chp.20001

[14] Project ECHO Replication Guide. Albuquerque, NM: ECHO Institute; 2016:1–21.

[15] Peterson R Darnell D Berliner L . Implementing transdiagnostic cognitive behavioral psychotherapy in adult public behavioral health: a pilot evaluation of the feasibility of the common elements treatment approach (CETA). J Behav Health Serv Res. 2018;46:249–266. 10.1007/s11414-018-9631-x 30209716

[16] Beidas RS Edmunds JM Cannuscio CC . Therapists perspectives on the effective elements of consultation following training. Adm Policy Ment Health. 2013;40:507–517. 23435832 10.1007/s10488-013-0475-7 PMC3676714

[17] Lyon AR Stirman SW Kerns SEU . Developing the mental health workforce: review and application of training approaches from multiple disciplines. Adm Policy Ment Health. 2010;38:238–253. 10.1007/s10488-010-0331-y PMC3093447 21190075

[18] Morrison AP Barratt S. What are the components of CBT for psychosis? A delphi study. Schizophrenia Bull. 2009;36:136–142. 10.1093/schbul/sbp118 PMC2800146 19880824

[19] Harris PA Taylor R Thielke R . Research electronic data capture (REDCap)—a metadata-driven methodology and workflow process for providing translational research informatics support. J Biomed Inform. 2009;42:377–381. 18929686 10.1016/j.jbi.2008.08.010 PMC2700030

[20] Harris PA Taylor R Minor BL The REDCap consortium: building an international community of software platform partners. J Biomed Inform. 2019;95:103208. 31078660 10.1016/j.jbi.2019.103208 PMC7254481

[21] RESOURCES: CBTp Competency Standards. British Columbia, Canada: North American CBT for Psychosis Network; 2020. Available from. https://www.nacbtp.org/resources. Accessed January 27, 2023.

[22] Kopelovich SL Basco M Stacy M . Position Statement on the Routine Administration of Cognitive Behavioral Therapy for Psychosis as the Standard of Care for Individuals Seeking Treatment for Psychosis. Alexandria, VA: National Association of State Mental Health Program Directors (NASMHPD) Publications. 2021. Available from. https://www.nasmhpd.org/sites/default/files/CBTp_Position_Statement_NASMHPD.pdf

[23] Rollinson R Smith B Steel C . Measuring adherence in CBT for psychosis: a psychometric analysis of an adherence scale. Behav Cogn Psychotherapy. 2008;36:163–178.

[24] Barch DM Bustillo J Gaebel W Logic and justification for dimensional assessment of symptoms and related clinical phenomena in psychosis: relevance to DSM-5. Schizophr Res. 2013;150:15–20. 23706415 10.1016/j.schres.2013.04.027

[25] Goldman HH Skodol AE Lave TR. Revising axis V for DSM-IV: a review of measures of social functioning. Am J Psychiatry. 1992;149:1148–1156. 1386964 10.1176/ajp.149.9.1148

[26] Kopelovich SL Strachan E Sivec H . Stepped care as an implementation and service delivery model for cognitive behavioral therapy for psychosis. Community Ment Health J. 2019;55:755–767. 30623294 10.1007/s10597-018-00365-6

[27] Project ECHO. Project ECHO Bibliography. Albuquerque, NM: University of New Mexico; 2016 Available from. https://digitalrepository.unm.edu/hsc_echo_bibliography/Accessed October 26, 2022.

[28] Johnson KL Hertz D Stobbe G Project extension for community healthcare outcomes (ECHO) in multiple sclerosis. Int J MS Care. 2017;19:283–289. 29270085 10.7224/1537-2073.2016-099 PMC5734711

[29] Baker RT Casanova MP Whitlock JN Expanding access to health care: evaluating project Extension for Community Health Care Outcomes (ECHO) Idaho's tele-education behavioral health program. J Rural Ment Health. 2020;44:205–216.

[30] Chaple MJ Freese TE Rutkowski BA Using ECHO clinics to promote capacity building in clinical supervision. Am J Prev Med. 2018;54 suppl 3:S275–S280. 29779552 10.1016/j.amepre.2018.01.015

[31] Cofta-Woerpel L Lam C Reitzel LR A tele-mentoring tobacco cessation case consultation and education model for healthcare providers in community mental health centers. Cogent Med. 2018;5:1430652. 30364535 10.1080/2331205X.2018.1430652 PMC6197484

[32] Paris M Hoge MA. Burnout in the mental health workforce: a review.J Behav Health Serv Res. 2009;37:519–528. 20013066 10.1007/s11414-009-9202-2

[33] Sivec HJ Montesano VL. Cognitive behavioral therapy for psychosis in clinical practice.Psychotherapy. 2012;49:258–270. 22642528 10.1037/a0028256

[34] Turner DT Burger S Smit F . What constitutes sufficient evidence for case formulation–driven cbt for psychosis? cumulative meta-analysis of the effect on hallucinations and delusions. Schizophrenia Bull. 2020;46:1072–1085. doi:10.1093/schbul/sbaa045 PMC7505201 32221536

[35] Routine Administration of Cognitive Behavioral Therapy for Psychosis as the Standard of Care for Individuals Seeking Treatment for Psychosis: State of the Science and Implementation Considerations for Key Stakeholders Acknowledgments. Rockville, MD: Substance Abuse and Mental Health Services Administration; 2021. Available from. https://store.samhsa.gov/sites/default/files/pep20-03-09-001.pdf. Accessed January 30, 2023.

[36] Kopelovich S Maura J Blank J . Sequential mixed method evaluation of the acceptability, feasibility, and appropriateness of cognitive behavioral therapy for psychosis stepped care. BMC Health Serv Res. 2022;22:1322. 36335319 10.1186/s12913-022-08725-5 PMC9636669

[37] Brabson LA Harris JL Lindhiem O . Workforce turnover in community behavioral health agencies in the USA: a systematic review with recommendations. Clin Child Fam Psychol Rev. 2020;23:297–315. 32103361 10.1007/s10567-020-00313-5

[38] Moullin JC Dickson KS Stadnick NA . Systematic review of the exploration, preparation, implementation, sustainment (EPIS) framework. Implement Sci. 2019;14:1. 30611302 10.1186/s13012-018-0842-6 PMC6321673

